# The Occupational Transition Process to Upper Secondary School, Further Education and/or Work in Sweden: As Described by Young Adults with Asperger Syndrome and Attention Deficit Hyperactivity Disorder

**DOI:** 10.1007/s10803-016-2986-z

**Published:** 2016-12-22

**Authors:** Vedrana Bolic Baric, Helena Hemmingsson, Kristina Hellberg, Anette Kjellberg

**Affiliations:** 1 Department of Social and Welfare Studies, Linköping University, Linköping, Sweden; 2 Department of Pedagogy, Linnaeus University, Växjö, Sweden

**Keywords:** Transition, Autism spectrum disorders, Employment, Education, Qualitative research, Services

## Abstract

The aim was to describe the occupational transition process to upper secondary school, further education and/or work, and to discover what support influences the process from the perspectives of young adults with Asperger syndrome or attention deficit/hyperactivity disorder. This qualitative study was performed in Sweden and comprised interviews with 15 young adults recruited from community based day centres. Support influencing the process included: occupational transition preparation in compulsory school, practical work experience in a safe environment, and support beyond the workplace. The overall understanding shows that the occupational transition process was a longitudinal one starting as early as in middle school, and continuing until the young adults obtained and were able to remain in employment or further education.

## Introduction

The transition is viewed in this study as an occupational transition process through which young adults leave compulsory school and undertake new challenges, associated with increased academic and work-related demands, including organizational and time management skills and social interaction (Frazier et al. 2007; Meaux et al. 2009; Pinder-Amaker 2014). The occupational transition from compulsory school to upper secondary school and further education and/or work is in this study regarded as a dynamic, interactional process between a person and the environment that involves changes in roles and expectations, and a range of choices (Christiansen and Townsend 2010; Shaw and Rudman 2009). The occupational transition involves daunting experiences for all young adults however, a growing body of research has demonstrated that both young adults with Asperger syndrome (AS) or attention deficit/hyperactivity disorder (ADHD) have a particularly difficult time becoming established in the labour market and in further education compared to those in the general population and persons with other disabilities including speech/language impairments and intellectual disabilities (Gerhardt and Lainer 2011; Taylor and Mailick 2014; Shifrin et al. 2010; Wei et al. 2014). AS and ADHD share an early onset, often co-exist and share overlapping symptoms including difficulties with social interaction, executive functions such as planning and organization, and comorbid conditions (Antshel et al. 2013; Mulligan et al. 2009; Van der Meer et al. 2012). A recent study showed that symptoms of AS and ADHD such as executive functioning and attention difficulties frequently co-occur and become increasingly intertwined over the course of development (Visser et al. 2016). Thus, due to overlapping difficulties and the similar patterns in reduced participation in further education and work, both individuals with AS and ADHD were included in the present study.

In Sweden, students with AS and ADHD commonly attend general education in compulsory school. The educational system in Sweden starts preparing students for the occupational transition to upper secondary school, employment and/or further education in the final years of their compulsory education (age about 15–16). Although general guidelines are available, access to transition services may vary between schools and municipalities (The Swedish National Agency for Education 2014). Following compulsory school, the Swedish State Employment Agency and the municipalities together offer vocational training through community-based day centres, or state-run labour market programmes to help young adults to find employment (The National Board of Health and Welfare 2009). Unemployment and a lower propensity to become involved in further education (Blasé et al. 2009; Shattuck et al. 2012) are particularly worrying given that unemployment is associated with low self-esteem, reduced health, and less community participation among young adults with disabilities, and also entails very high costs for national economies (The Ministry of Education and Research 2013).

Studies about the occupational transition to upper secondary school, further education or employment for young adults with AS or ADHD only concerns parts of the occupational transition process. Research has either focused on decision-making and preparation for the upcoming occupational transition (Mitchell and Beresford 2014) or transition programs implemented in compulsory school, as parts of the process (Hagner et al. 2012; Griffin et al. 2013; Strickland et al. 2013; Shandra and Hogan 2008). Studies have also focused on how to help young adults with AS or ADHD to continue at university (Hagner et al. 2014; Lee et al. 2008; Lee and Carter 2012; Madariaga et al. 2008; Meaux et al. 2009; van Hees et al. 2015), or in employment, as part of the process (Burke-Miller et al. 2012; Howlin et al. 2005; Nicholas et al. 2015; Wehman et al. 2012). A few studies have applied the perspective of young adults with AS or ADHD (Madariaga et al. 2008; Meaux et al. 2009; Mitchell and Beresford 2014; van Hees et al. 2015). One study (Mitchell and Beresford 2014) has focused on how young adults with AS experience the early part of the transition process, as they make decisions about, and prepare for the occupational transition to a further educational setting. In these findings, parents providing emotional support, organizing meetings and college visits, and communicating support needs were all highlighted as beneficial for preparation. Similarly, Madariaga et al. (2008) has focused on the lives of students with AS during their first year in higher education. This study particularly stressed the euphoria students with AS expressed throughout their first academic year, as well as the opportunity to place negative experiences of school, such as social isolation and bullying, behind them. Another study exploring how young adults with ADHD experience the later part of the occupational transition process showed that academic support from mentors and academic disability services, and support in managing everyday life from peers, influenced whether these young adults remained at university (Meaux et al. 2009). Van Hees et al. (2015) also focused on those students that are enrolled in college reporting on the struggles students’ face in college, including difficulties with new situations and unexpected changes, social relationships, problems with information processing and time management and mental health. The students’ emphasised the need for a personalized approach, academic accommodations, coaching in education, student life and daily living, psychosocial support and leisure activities. Thus, previous research demonstrates the need to explore how young adults with AS or ADHD experience the entire occupational transition process to upper secondary school, further education and/or employment, including preparation and decision-making following compulsory school, gaining employment or enrolling in further education, as well as remaining in employment or further education. To be better able to meet young adults’ with AS and ADHD need for support in all parts of the occupational transition process to upper secondary school, further education and working life, knowledge about their experiences and thoughts during the transition process is urgently required (Test et al. 2014).

## Aim

To describe the occupational transition process to upper secondary school, further education and/or work and to explore what support influences the process from the perspectives of young adults with AS or ADHD.

## Materials and Methods

The study had a qualitative design and was based on interviews that were analysed with a hermeneutical approach (Gadamer 2004). It focuses on interpretation and assumes that all understanding is in itself always a matter of interpretation. According to Gadamer (2004), all understanding involves the realm of pre-understandings which are viewed as enabling analysis by providing a starting point for understanding of people’s experiences. The first author had experience of working within health care and with occupational therapy education. From an occupational therapy perspective the interplay between people, their occupations and roles, and the environments in which they work and/or study is crucial for understanding the occupational transition from compulsory school to upper secondary school, further education and/or work. These pre-understandings are seen as influencing the interpretation. Ethical approval was granted by the Regional Ethics Committee in Linköping, Sweden Dnr 2010/292-31.

### Recruitment of Participants

Potential participants were identified by staff members at three municipality services responsible for providing support and services to the target group in the mid-east region of Sweden. Senior administrators at each municipality service identified potential participants based on the inclusion criteria, and approached staff members such as coaches at community-based day centres providing work experience through individual placements in regular workplaces or staff at group housing in the participants’ immediate environment to inform them about the study, both orally and through written communication. A purposeful sampling (Patton 2015) was used based on the following inclusion criteria: young adults between the ages of 18 and 30 years, with a primary diagnosis of AS or ADHD, based on the Diagnostic and Statistical Manual of Mental Disorders (4th ed.; DSM-IV^1^) and/or International Classification of Diseases, 10th Revision (ICD-10) (American Psychiatric Association 2000; World Health Organisation 1993), and willing and able to communicate their experience of the occupational transition process to upper secondary school, further education and work. Participants willing to participate in the study were asked for permission by staff members for the first author to contact them. The ability to perform an interview was assessed by staff members. Details of only those participants who gave consent to participate in the study were passed to the first author. Time and place for the interviews were scheduled together with the young adults and performed by the first author in places that were convenient and chosen by the participants. In total, 17 interviews were performed. Based on the inclusion criteria, two participants were excluded due to not having a formal diagnosis of AS or ADHD, giving a total sample of 15 participants in the present study.

### Participants

Of the 15 young adults, 10 had been diagnosed with AS and five with ADHD. The ages of the participants ranged from 20 to 29 years, and eight men and seven women participated. For information concerning the participants’ highest level of education and current employment or education status see Table 1. Six of the participants received their diagnosis in the early years of compulsory school, with the rest of the participants receiving their diagnosis after graduating from compulsory school or upper secondary school.

**Table 1:** Demographic information about the participants

Participants	Sex		Age (years)	Diagnosis	Receiving the diagnosis during/after compulsory school	Highest level of education completed	Current employment and educational status	Transition path
								A straightforward occupational transition to community-based day centres
Martina	F	20		AS	During compulsory school	Mainstream upper secondary education	Community-based day centre^a^	⇒ community-based day centre
David	M	20		ADHD	After compulsory school	Upper secondary school for individuals with intellectual disabilities	Community-based day centre	⇒ community-based day centre
Adam	M	21		AS	After compulsory school	Mainstream upper secondary education	Community-based day centre	⇒ community-based day centre
Emily	F	22		AS	After compulsory school	Mainstream upper secondary education	Community-based day centre	⇒ community-based day centre
Sandra	F	22		ADHD	During compulsory school	Compulsory education	Community-based day centre	⇒ dropped out of upper secondary school for individuals with intellectual disabilities ⇒ community-based day centre
								Interrupted periods of adult education, community-based day centres and work in the regular labour market
Renée	F	21		ADHD	After compulsory school	Compulsory education	Folk high school^b^	⇒ introductory programme for pupils who are not eligible for a national programme ⇒ community-based day centre ⇒ dropped out of mainstream upper secondary school ⇒ folk high school
Sebastian	M	22		AS	After compulsory school	Mainstream upper secondary education	Community-based day centre	⇒ folk high school ⇒ work experience in the regular labour market ⇒ community-based day centre
Tom	M	23		AS	After compulsory school	Compulsory education	Community-based day centre	⇒ dropped out of mainstream upper secondary school ⇒ introductory programme for pupils who are not eligible for a national programme ⇒ folk high school ⇒ work experience at the regular labour market ⇒ community-based day centre
Jenny	F	24		AS	After compulsory school	Compulsory education	Community-based day centre	⇒ work experience in the regular labour market ⇒ community-based day centre
Carl	M	24		ADHD	During compulsory school	Dropped out of compulsory school	Community-based day centre	⇒ work experience in the regular labour market ⇒ community-based day centre
Catharina	F	28		ADHD	After compulsory school	Dropped out of compulsory school	Folk high school	⇒ community-based day centre ⇒ folk high school
Jonas	M	29		AS	After compulsory school	Mainstream upper secondary education	Community-based day centre	⇒ folk high school ⇒ work experience in the regular labour market ⇒ community-based day centre
Paul	M	28		AS	During compulsory school	Upper secondary school for individuals with intellectual disabilities	Community-based day centre	⇒ folk high school ⇒ community-based day centre
								Occupational transition to university studies and employment in the regular labour market
Jonathan	M	23		AS	During compulsory school	Individually adapted upper secondary school programme	Probationary employment^c^	⇒ university studies ⇒ community-based day centre ⇒ regular labour market
Mary	F	28		AS	After compulsory school	Mainstream upper secondary education	Labour market programs^d^	⇒ work experience in the regular labour market ⇒ university studies ⇒ community-based day centre

^a^Traditionally, day centres have been meeting place-oriented or work-oriented, situated in one building (Tjörnstrand et al. 2011). The trend has been towards more flexible forms outside the normal day centres e.g. work experience through individual placements in a regular workplace

^b^Folk high schools are independent adult education colleges with courses that can be oriented towards individuals with AS and ADHD

^c^A probationary period of employment for a maximum period of six months in which the employee has the opportunity to learn the job, while the supervisor can observe and evaluate the employee’s performance

^d^Labour market programmes provide different measures targeting the unemployed and people who are remote from the labour market

### Data Collection

The interviews were performed by VBB using a semi-structured interview guide consisting of broad, open-ended questions that covered the following: (1) current situation in relation to education and work, (2) the occupational transition process to upper secondary school, further education and work, and (3) experience of support that facilitated and/or constrained the occupational transition process to upper secondary school, further education and work. The participants were for example asked to describe their experience throughout the whole occupational transition process, from graduating from compulsory school to continuing on to further education and/or work, as well future aspirations. Follow-up questions, probes and the young adults’ vocabulary were used when needed in order to help the participants to develop their descriptions, as suggested by Harrington et al. (2013). A timeline (Polit and Beck 2008) was used together with the participants to sort their experiences into chronological order from compulsory school to their current situation. The interviews lasted between 1 and 2.5 h, with half lasting longer than 1.5 h. The interviews were digitally recorded and transcribed verbatim.

### Data Analysis

Each interview text was initially read and re-read several times by all authors in order to develop an overall understanding of what the participants were describing (Gadamer 2004). In the subsequent in-depth re-reading of the transcripts, segments of text were extracted by the first author that provided insight into the participants’ experience of the occupational transition process to upper secondary school, further education and work, and support that influenced the process. In the next step of the analysis, in line with Gadamer (2004) the transcripts were re-read several times as a whole, after which the first author actively engaged in a dialogue with the text by posing questions to the text, including the following: How did the participant describe their experience of the occupational transition process? What “stages” does the occupational transition process to upper secondary school, further education and/or work entail? During which “stages” did the participants describe receiving support? In this step all authors were involved in the analysis in order to critically question the first author’s understanding of the text, and in the search for alternative explanations. Next, in order to explore the occupational transition process to upper secondary school, further education and/or work, the timeline was used by the first author as an analytic tool to display the participants’ transition pathways following compulsory school. By sequencing the participants’ pathways in a chronological order from compulsory school to current educational and/or further education situation, as part of the analysis, three different pathways could be identified and labelled; “a straightforward occupational transition to community-based day centres”; “interrupted periods of adult education, community-based day centres and work in the regular labour market” and “occupational transition to university studies and employment in the regular labour market”. Different characteristics describing the identified pathways were noted. Next, in order to describe support that influenced the occupational transition process, participants’ experiences of received support and support needs were compared with each other by searching for patterns as well as overlaps and contrasts. Similar experiences were clustered together in different categories, referred to as sub-clusters. The identified sub-clusters were; “occupational transition preparation in compulsory school”; “practical work experience in a safe environment” and “support beyond the workplace”. Next, sub-clusters were compared with each other through a process of repeatedly moving between the transcripts and the sub-clusters. After this, similar experiences of support influencing the occupational transition process were grouped together in two overarching themes, referred to as clusters. These clusters were labelled “occupational transition paths to upper secondary school, further education and/or work” and “experience of support that influenced the occupational transition”. In this step all authors were involved in the search for alternative clusters by comparing similarities and dissimilarities in experiences. This was performed by continually moving between the transcripts, emerging sub-clusters and clusters with the intention of ensuring that the interpretations were grounded in the data. The understanding of the experiences of support was continuously discussed among all authors and clusters were modified as a result. Finally, in order to achieve a comprehensive understanding, the three identified transition paths were analysed in relation to the three sub-clusters. This resulted in similarities and dissimilarities emerging between different paths and experiences of support. The last step involved checking different interpretations against the clusters and sub-clusters in order to achieve an overall interpretation that bound all parts of the data together (Gadamer 2004). All four authors were involved in discussions in the search for different interpretations that covered all parts of the data. One interpretation concerning support that influenced the occupational transition process was linked to receiving a medical diagnosis while in compulsory school. This was checked against the data. In addition, it was also checked whether individuals with AS differed in their transition pathways or in their experience of support compared to those with ADHD. No such differences were grounded in relation to the data. In further analysis, an interpretation related to viewing the occupational transition process as a longitudinal process between the person and the environment emerged as the most fruitful for an overall understanding of clusters, sub-clusters and their relationships.

### Establishing Trustworthiness

In qualitative research, the concepts of credibility, transferability, dependability and confirmability, are used to illustrate different aspects of trustworthiness. Gadamer (2004) uses the concept of valid interpretations, which is applicable to the criteria used for trustworthiness (Fleming et al. 2003). Credibility was attained by involving all the co-authors in the following steps of the analysis; (1) in discussions of the overall understanding of the text, (2) critically questioning the first author’s understanding of the text, and (3) in the search for alternative explanations, sub- clusters, clusters and overall understanding (Krefting 1991; Patton 2015; Polit and Beck 2008). Detailed descriptions are provided of the selection of participants, characteristics of the participants, the data collection and the process of analysis, with the intention of enabling the reader to assess how transferable the results are to other contexts (Patton 2015; Polit and Beck 2008). The names of the participants have been altered in the study to ensure confidentiality. A reflexive diary was used as means to enhance the dependability of qualitative research (Patton 2015). Initial expectations that the first author was consciously aware of were written down and reflected upon, as well as changes in the author’s pre-understandings that developed over time. Confirmability was attained by using the timeline, enabling participants to immediately provide feedback on the first author’s initial interpretations (Krefting 1991; Patton 2015).

## Results

The results will be presented in two sections. The first section describes three different pathways that the participants followed to upper secondary school, employment and/or further education (see Table 2). The second section describes experiences of support and support needs during the occupational transition process (see Table 3).

**Table 2:** Overview of clusters and sub-clusters in relation to occupational transition pathways

Cluster	Occupational transition pathways		
Sub-cluster	A straightforward occupational transition to community-based day centres	Interrupted periods of adult education, community-based day centres and work in the regular labour market	Occupational transition to university studies and employment in the regular labour market

**Table 3:** Overview of clusters and sub-clusters in relation to experience of support that influenced the occupational transition

Cluster	Experience of support that influenced the occupational transition		
Sub-cluster	Occupational transition preparation in compulsory school	Practical work experience in a safe environment	Support beyond the workplace

### Occupational Transition Paths to Upper Secondary School, Further Education and/or Work

The participants’ pathways showed three different patterns (see Table 1): (1) those who made a straightforward occupational transition to community-based day centres; (2) those who “tried out” a variety of employment and further education alternatives, followed by community-based day centres; and (3) those who made the occupational transition to university studies and employment in the regular labour market.

### A Straightforward Occupational Transition to Community-Based Day Centres

The first pathway was characterized by a straightforward pathway from mainstream upper secondary school or upper secondary school for individuals with intellectual disabilities to current participation in community-based day centres at local businesses including coffee shops, cafes or carpenter’s jobs. Participants (n = 5) following this pathway were aged 22 or younger and all except for one had completed education at an mainstream upper secondary school, or upper secondary school for individuals with intellectual disabilities (see Table 1). These participants attended both vocational programmes and further education preparatory programmes at upper secondary school. Two of the participants had received their diagnosis during their compulsory school education. This pathway was characterized by clear aspirations of finding employment in the regular labour market following compulsory school. Employment was for these young adults closely connected to financial security, rather than dreams and aspirations of working with specific careers; “I am a person who is satisfied with just having a job” (Adam). This pathway was further characterized by young adults embarking on the clearly marked path to the Swedish State Employment Agency for finding employment, followed by early support received through community-based day centres. These participants had difficulties finding a job in the regular labour market and were aware of their need for additional support due to problems including stress and issues with structuring and planning activities; “I knew that it would be hard to find a job if you have Aspergers. A lot of jobs requires you to handle stress, and I don’t think that fits in with me” (Martina). Participants also expressed a striking confidence in their ability to progress towards employment in the regular labour market with support from the community-based day centres.

### Interrupted Periods of Adult Education, Community-Based Day Centres and Work in the Regular Labour Market

This pathway was characterized by young adults (n = 8) trying out a variety of options following compulsory school, with repeated failure to find and remain in employment and further education. The majority of these participants were >24 years old and many lacked upper secondary school education. Many participants described having to supplement either their compulsory or upper secondary school education by municipal adult education. Those who had been to upper secondary school attended vocational programmes. This pathway was characterized by both unclear aspirations and transition goals regarding what these participants wanted to do following compulsory school or what upper secondary school programme to attend, as well as aspirations being thrown into question. Three of these participants received their diagnosis while in school. This pathway was also characterized by young adults trying out a variety of employment or further education options, as well as work experiences in the regular labour market, in an effort to find an option that was suitable for them, as shown in Table 1. Furthermore, this pathway was characterized by challenges in meeting work and further education demands, as illustrated by Sebastian; *“*It did not work out well enough for me to be able to keep on working at the bakery. ‘I was not good enough to be able to keep a job there’. No, not a regular employment”. Lack of support in earlier workplaces, and support decreasing over time were also mentioned by the participants. In particular, these participants displayed difficulties with emotional wellbeing, including anxiety and stress, and many explicitly stated they needed support from the health care system for their emotional wellbeing, as illustrated by Jonas; “I explained multiple times that I have experienced emotional distress during lot’s of years and so, only to receive occasional visits with a psychologist. Of course I feel that I would have benefitted from having had someone to talk to during all of these years”. Failure keeping jobs or dropping out of further education threw their future aspirations into question, and many had to find new ways of thinking about their future. They dealt with this by changing their future aspirations, by reducing their expectations of finding jobs in the regular labour market in the near future, or by giving up on certain careers.

### Occupational Transition to University Studies and Employment in the Regular Labour Market

Two participants made the transition to university studies and employment in the regular labour market, but without graduating. This pathway was characterized by clear aspirations of attending university as a means to employment. These participants attended a preparatory programme at upper secondary school (see Table 1). One of them had received the diagnosis while in compulsory school. Attending university was closely tied to a sense of an ordinary life, which was something that they strived for, as expressed by Mary; “I have always struggled with not being worthless and bad, and have really made an effort to be normal”. These participants displayed confidence in their own abilities to graduate from university and find a job in the open labour market. Despite experiencing difficulties managing the course load at university, these participants believed that they would obtain a job in the open labour market; “Life after upper secondary school does not have to be awful. There is a future for everyone if you just want it. When I was 16 I knew that I wanted to work with accounting since math was my favourite subject and that is what I choose to study at the university” (Jonathan). This conviction was shared and encouraged by their parents, to whom they looked predominantly for support.

### Experience of Support that Influenced the Occupational Transition

In this section, support that influenced the process to upper secondary school, further education and work will be set out under the three sub-clusters, with differences between the support that influences the process under the three pathways being identified.

### Occupational Transition Preparation in Compulsory School

Transition preparation typically began while the participants were close to graduating from compulsory school, and involved them engaging in a range of preparatory activities in order to prepare them to make educational and/or work-related choices. Participants expressed different support needs and gave examples of what concretely needed to be done, according to the different transition paths. In particular, participants who followed the second pathway, without clear goals for what they wanted to do following compulsory school, consistently said that visit days and information from other students and education advisors were introduced too close to graduation from compulsory school, and as such, did not allow sufficient time for preparation. The need for early planning was described by Tom:

“For example in order to find an upper secondary school programme, having more days for trying out would be beneficial. A longer period to search for schools, explore interests, what one likes and what one wants to do. To watch movies. It is important to choose correctly. This you need to do already in seventh grade so that you have 2–3 years to look up what grades need to be attained before graduation.”

Participants who followed the second pathway stressed the need for parents, as well as teachers to be responsive to the participants’ dreams and aspirations in the early stages of compulsory school. Participants provided concrete examples of what parents and teachers could do, including providing encouragement and support to explore interests, as well as information on careers that matched their interests and background, and the education needed for the identified career.

Participants who followed the first and third pathways were characterized by clear career aspirations and transition goals. In contrast to participants following the second pathway, these participants’ appreciated meetings with education advisors in close connection to graduation from compulsory school. However, analysis revealed that these meetings need to be based on the student’s unique strengths and interests in order for the participants to choose an alternative in upper secondary school, further education or work, which matched their interests and abilities, as illustrated by Emily; “I would have benefitted from support in choosing something that suited me better. Well like maybe to sit down with someone (school personnel) before and go through what was available to choose from and what that means, for example, what does that upper secondary school programme entail”. Furthermore, the five young adults who received their diagnosis while in compulsory school did not describe different transition preparation experiences or support from professionals compared to those who received their diagnosis after completing compulsory school.

### Practical Work Experience in a Safe Environment

Participants following the first and second pathways after compulsory school emphasized that community-based day centres consisting of individual placements at local businesses, with on-the-job support provided by staff members were highly influential for making a gradual labour market entry by providing participants with practical work skills and building up their curriculum vitae, which was necessary to ensure future employment opportunities. Rather than looking upon community-based day centres as a barrier to their ultimate goal of finding employment in the regular labour market, all of these participants emphasized possibilities following attendance at community-based day centres, including more chances of eventually finding a job that was right for them; “Getting the opportunity to work may end up causing me to finally find something that suits me, making me get a real job” (Sebastian). Participants who followed the first pathway considered that community-based day centres supported the development of practical work skills such as time management, and learning about one’s own capacities, resulting in a growing sense of confidence. Participants who followed the second pathway with experience of repeated failure highlighted that community-based day centres created a safe environment in which they had room to fail and learn from their mistakes, as illustrated by Tom’s description of community-based day centres:

“Not feeling that you have to do great from the beginning, that you are able to fail, that you get space to test yourself and see, and if you get it wrong than you get it wrong, it’s not the end of the world and one should learn from mistakes”.

These participants emphasized the need for recurring and long-term support over several years in the workplace in order to remain in employment. The importance of close and trusting relationships with professionals from the community-based day centres was stressed by participants following the first and second pathways.

On the other hand, participants that followed the third pathway and those with previous work experience in the regular labour market following the second pathway said that community-based day centres made them feel too remote from the labour market. These participants said that attending community-based day centres, even if it was for a short period, made their difficulty finding a job visible to others, with the result that their ability to maintain a job might be questioned.

### Support Beyond the Workplace

Regardless of the transition path, participants said that their whole life situation, including residential, economical and emotional aspects had influenced whether or not they could remain in employment. Receiving practical support beyond the workplace was crucial for maintaining attendance at community-based day centres: “I need support in getting to community-based day centres, to make sure that you get there” (Emily). Being unable to pursue their daily routines and habits, degraded their emotional wellbeing, which in the long run influenced their motivation and energy for work: “I need support with mountains of dishes, clothes, dirty laundry, bills, yes everything that piles up at home. When it has built up over a long time, you do not know which end to start with” (Jonas). For participants following the first and second pathways, practical support was usually provided by various staff members in the participants’ homes. Participants following the third pathway highlighted that parents and significant others, e.g. friends or partners, usually provided them with practical support in their homes.

### Longitudinal Process with Support from Different Professionals and Family Members

The findings indicate that the occupational transition process towards upper secondary school, employment or further education was described as a longitudinal process starting as early as middle school and continuing to when the subjects were young adults, and it included obtaining and remaining in employment or further education. The occupational transition process was influenced not only by support, but by the interaction between environmental support and personal factors. Personal factors, including difficulties with emotional wellbeing, were described as hindering the process, while having clear transition goals facilitated the process. Environmental support such as receiving support from community-based day centres was described by some participants as important for gaining employment. In addition, various services, ranging from the educational system to employment services and health care and significant others (e.g. parents and partners) supporting home-based activities were also described as crucial for enabling the process.

## Discussion

The current study adds to the underexplored research area on the entire occupational transition process from the perspective of young adults with AS or ADHD. Previous studies have provided knowledge on the different parts of the occupational transition process including decision-making and preparing for the occupational transition (Mitchell and Beresford 2014) and remaining in employment or further education (Meaux et al. 2009). The present study’s findings revealed that the occupational transition process towards upper secondary school, employment or further education may be considered as a longitudinal process that begins with preparedness in compulsory school, moves on to enrolment in upper secondary school or further education or obtaining employment, and culminates in remaining in employment or further education. Furthermore, the progression may be considered as an interactive process between personal factors and environmental support (Christiansen and Townsend 2010; Shaw and Rudman 2009).

The findings revealed that most of the young adults with AS or ADHD with difficulties obtaining and remaining in employment received services through community-based day centres (i.e. environmental support). Community-based day centres as described by the participants in the present study included work experience through individual placements at local businesses, with on-the-job support provided by staff members, which may be comparable to supported employment, comprising of a rapid job search, community placement and job coaching (Nicholas et al. 2015). Findings revealed that community based day centres were described by all young adults with AS and ADHD following the first and second pathway as fostering a sense of hopefulness and motivation towards finding employment in the regular labour market, indicating that this type of placement facilitates the progression to the regular labour market. However, this needs further research. This finding is in contrast to the concerns raised by The Swedish National Board of Health and Welfare (2008) about the “lock-in effects” of day centres, whereby those who come to day centres tend to remain there without transitioning to paid employment. Similarly, earlier studies have highlighted the need for day centres to provide channels to jobs on the open labour market through collaboration with local businesses and the surrounding community beyond the day centres in order to facilitate engagement in employment in the open labour market (Eklund and Sandlund 2014; Eklund and Tjörnstrand 2013; Tjörnstrand et al. 2015). Findings also showed that participants with AS or ADHD with experience of occupational transition to university and employment in the regular labour market described community-based day centres as being far away from the labour market, even if they received individual placements in a regular workplace. The risk of participants feeling separated and alienated from the society when participating in community based day centres has been previously reported for adults with psychiatric disabilities (Bryant et al. 2004; Pinfold 2000). Further research is warranted to explore the longitudinal pathways for young adults with AS or ADHD following community based day centres attendance.

Even though our findings showed no differences between young adults with AS and ADHD in regards to experiences of support for the occupational transition process, the question whether the specific characteristics of AS or ADHD influence the occupational transition process can be discussed. Findings revealed that young adults with AS following the first and second pathway experienced difficulties with executive functioning including planning, organization and time management in further education and/or work. These challenges correspond to the developmental and behavioral aspects of AS, which are characterized by weak central coherence, difficulties with theory of mind and difficulties related to executive functioning (Volkmar and Wiesner 2009). Difficulties with organization and planning have been reported as important aspects of the occupational transition process (Langberg et al. 2013; VanBergeijk et al. 2008). The findings highlight the positive contribution of practical work experience provided by local businesses in supporting young adults with AS to develop practical work skills such as time management. This finding is consistent with earlier studies reporting on the positive effects of engagement in real work settings in improving executive functioning among individuals with autism (García-Villamisar and Hughes 2007). Further research is warranted to determine the possible effect of work on executive functioning among individuals with AS. Furthermore, the findings revealed that young adults with AS felt unprepared for the occupational transition, and struggled with stress and anxiety. Stress and anxiety have been linked to the core features of AS (American Psychiatric Association 2013). As such, the findings confirm that young adults with AS are inadequately prepared for the occupational transition process (e.g. Mitchell and Beresford 2014; Wiesner et al. 2009) and correspond with findings that show that the occupational transition to upper secondary school may exacerbate levels of stress and anxiety (Fortuna 2014). In order to prepare for the occupational transition process and as such prevent stress and anxiety, young adults with AS pointed to the importance of clear information and guidance from teachers, education advisors and parents and preparing a head of time for the occupational transition. They also stressed the need for emotional support from professionals, as well as support from professionals at the workplace.

In particular, young adults with AS and ADHD without clear transition goals related to employment or further education described trying out a variety of educational or further education options, followed by difficulties obtaining and remaining in employment or further education. Having clear post-upper secondary school goals has been identified as a significant factor associated with participation in employment for young adults with autism (Chiang et al. 2013). Support for the development of transition goals needs to be implemented in the early transition planning in school. Kohler and Field (2003) recommended early transition preparation in compulsory school, not as specific activities for students with disabilities once they reach the age of 14 or 16 years, but rather as a transition-focused process throughout compulsory school, delivered through a variety of transition activities and by a range of services based on students’ needs. Further research is warranted in order to understand what preparatory transitional activities and services implemented during compulsory school support the move to obtaining employment and enrolling in further education.

### Practical Implications

The findings point to the need for teachers’, education advisors’ and parents’ to consider the transition process as a longitudinal process starting as early as middle school and continuing on until young adults with AS or ADHD obtain employment or remain in further education. The findings highlight the need for those working with young adults with AS or ADHD to develop an individualized transition planning as well as providing them with guidance and support throughout the occupational transition process. Several key elements were described by the young adults as supporting an individualized transition planning including; teachers, parents and education advisors; (1) supporting students to define their own transition goals; (2) support in exploring interests, (3) providing information on careers that matched student’s interests, as well as information on qualifications required to pursue the desired careers. The findings also call for inter-service collaboration between students, parents, schools, employment services and the health care system in order to ensure that critical occupational transition needs are met. Furthermore, the findings revealed that community based day centers consisting of individual placements in a regular workplace with on-the-job support provided by professionals can be considered as having positive influences on the occupation transition process to further education and/or work.

## Limitations

The results of this study should be interpreted with the following limitations in mind. The participants were all recruited from different community-based municipality services, and therefore the findings may not be generalizable to young adults without any support services. Consequently the results cannot be generalized to individuals employed in the regular labour market or studying at university. The results cannot be generalized to young adults with other disabilities either. The diagnosis of AS and ADHD was confirmed on information based on medical records obtained from staff members. Even if the staff members were convincing when they reported the medical information some caution should be applied in interpreting the data. The use of qualitative research in this study aims at describing solely the participants’ experiences, and does not attempt to describe parents’ or teachers’ experiences of the occupational transition process. Having included these groups as well in the present study had given a multifaceted perspective on the occupational transition process. Interviewing young adults with AS and ADHD may be a challenge due to the speech, language, and communication difficulties associated with the conditions. However, the semi-structured interview guide used supported using flexible strategies, such as probes in order to adapt the interview to each young adults’ own abilities when necessary (Harrington et al. 2013).

## Footnotes

1 In the Diagnostic and Statistical Manual of Mental Disorders (DSM-5) Asperger’s disorder, is situated under the umbrella disorder Autism Spectrum Disorder (American Psychiatric Association 2013).
